# Positive association of tomato consumption with serum urate: support for tomato consumption as an anecdotal trigger of gout flares

**DOI:** 10.1186/s12891-015-0661-8

**Published:** 2015-08-19

**Authors:** Tanya J Flynn, Murray Cadzow, Nicola Dalbeth, Peter B Jones, Lisa K Stamp, Jennie Harré Hindmarsh, Alwyn S Todd, Robert J Walker, Ruth Topless, Tony R Merriman

**Affiliations:** Department of Biochemistry, University of Otago, Box 56, Dunedin, New Zealand; Department of Medicine, University of Auckland, Auckland, New Zealand; Department of Medicine, University of Otago, Christchurch, New Zealand; Ngati Porou Hauora Charitable Trust, Te Puia Springs, New Zealand; Mater Research Institute, Brisbane, Australia and School of Allied Health Sciences, Griffith University, Gold Coast, Australia; Department of Medicine, University of Otago, Dunedin, New Zealand

## Abstract

**Background:**

Gout is a consequence of an innate immune reaction to monosodium urate crystals deposited in joints. Acute gout attacks can be triggered by dietary factors that are themselves associated with serum urate levels. Tomato consumption is an anecdotal trigger of gout flares. This study aimed to measure the frequency of tomato consumption as a self-reported trigger of gout attacks in a large New Zealand sample set, and to test the hypothesis that tomato consumption is associated with serum urate levels.

**Methods:**

Two thousand fifty one New Zealanders (of Māori, Pacific Island, European or other ancestry) with clinically-ascertained gout were asked about gout trigger foods. European individuals from the Atherosclerosis Risk In Communities (ARIC; *n* = 7517) Study, Cardiovascular Health Study (CHS; *n* = 2151) and Framingham Heart Study (FHS; *n* = 3052) were used to test, in multivariate-adjusted analyses, for association between serum urate and tomato intake.

**Results:**

Seventy one percent of people with gout reported having ≥1 gout trigger food. Of these 20 % specifically mentioned tomatoes, the 4^th^ most commonly reported trigger food. There was association between tomato intake and serum urate levels in the ARIC, CHS and FHS combined cohort (β = 0.66 μmolL^−1^ increase in serum urate per additional serve per week; *P* = 0.006) - evident in both sexes (men: β = 0.84 μmolL^−1^, *P* = 0.035; women: β = 0.59 μmolL ^−1^, *P* = 0.041).

**Conclusions:**

While our descriptive and observational data are unable to support the claim that tomato consumption is a trigger of gout attacks, the positive association between tomato consumption and serum urate levels suggests that the self-reporting of tomatoes as a dietary trigger by people with gout has a biological basis.

**Electronic supplementary material:**

The online version of this article (doi:10.1186/s12891-015-0661-8) contains supplementary material, which is available to authorized users.

## Background

High serum urate levels are the major risk factor for gout [1]. Urate levels are maintained within the body through a balance between urate production (hepatic) and excretion (renal and gut), controlled by genetic and dietary factors [2]. When urate levels reach supersaturation monosodium urate crystals may deposit within the joints and elicit an immune reaction [3]. Variants in genes encoding renal and gut uric acid transporters are associated with urate levels and gout [4–8], while serum urate-controlling variants of weaker effect map to loci thought to be involved in glycolysis and other pathways enriched for inhibins and activins [5].

Foods and beverages positively associated with serum urate and gout are alcohol (particularly beer), purine-rich foods, red meat, seafood, and sugar-sweetened beverages [9–15]. Conversely, intake of coffee, dairy products and vitamin C have been associated with lowered serum urate levels and reduced risk of gout [11, 16–20]. Urate is generated from breakdown of purines and as a consequence of hepatic metabolism of alcohol and sugar, with evidence that both ethanol and sugar also interfere with renal excretion of uric acid [9, 21]. Gout is often associated with dietary triggers, commonly perceived by patients to be the most important cause of gout [22]. Demonstrated food triggers, identified using case-crossover study design, include alcohol and purine-rich foods [23, 24]. These foods also increase serum urate levels [12, 15, 25], consistent with the hypothesis that they trigger acute gout attacks. People with gout also self-report food avoidances that have not been substantiated by the medical literature – these avoidances include tomatoes and tomato products [26].

This study aimed to determine the frequency of tomato consumption as a self-reported trigger of gout flares in a large gout sample set from New Zealand (including Māori and Pacific Island participants). These groups have a prevalence of gout double that of European Caucasian [27], with earlier onset, more severe gout presentation and a higher prevalence of co-morbidities [28]. We also tested the hypothesis that tomato consumption is associated with serum urate levels.

## Methods

New Zealand men and women >17 years of age with gout (*n* = 1791) (recruited as part of a study focused on the risk factors for gout) [29] were asked the question “Do certain foods/drinks trigger your gout?” Patients were prompted to specify whether alcohol or seafood triggered their gout, then given the opportunity to list other gout trigger foods/drinks in an open-question format. Responses to the same question by a separate Māori sample set from the rohe (area) of the Ngati Porou iwi (tribe), patients of the primary health care provider Ngati Porou Hauora (health) Charitable Trust, in the Tairawhiti region on the East Coast of the North Island of New Zealand were also analysed (260 people with gout). All participants had a confirmed diagnosis of gout, as defined by the 1977 American Rheumatology Association preliminary classification criteria for acute gout [30]. They were recruited from community-based settings, and primary and secondary health care. Ethical approval was given by the New Zealand Multi-Region and Northern Y Ethics Committees and all participants provided written informed consent for the collection of samples and subsequent analysis. Anthropometric, clinical and gout attack trigger information for each analysis cohort are contained in Additional file 1: Table S1.

Trigger foods were summarised into ten categories (alcohol, dairy products, fruit, poultry, red meat, seafood/fish, sugar-sweetened beverages, tomatoes, vegetables, and other) and the percentage of people who mentioned each category calculated – split also into five ancestral groups, New Zealand Māori, Ngati Porou Māori, New Zealand Pacific Island, European and Other. Multivariate logistic regression adjusted for sex, age at first attack, body mass index, and number of flares per year was conducted to determine whether ancestry influenced the reporting of acute gout attack triggers.

Data from the Atherosclerosis Risk in Communities (ARIC) (http://www2.cscc.unc.edu/aric/), Cardiovascular Health Study (CHS) (https://chs-nhlbi.org/) and Framingham Heart Study (FHS) Generation 3 (http://www.framinghamheartstudy.org/) cohorts were used to test the hypothesis that tomato consumption is associated with serum urate levels. All three studies recruited individuals from North America. Participants in these studies completed a food frequency questionnaire that asked “How often, on average, did you eat tomatoes or tomato juice in the past year?” (Tomato sauce information was also collected, but excluded for this analysis). The questionnaire also asked about consumption of seafood/fish (canned tuna fish, dark meat fish, shrimp, lobster, scallops, and other fish), red meat (beef, pork, lamb, bacon, processed meat, hamburgers, and hotdogs), alcohol (beer, wine, and liquor), sugar-sweetened drinks (regular soft drink and non-carbonated fruit drink), coffee (not decaffeinated) and dairy products (milk, cream, butter, margarine, ice cream, cheese and yoghurt). Vitamin C intake (mg/day) was also measured in each of these studies. Study participants were asked to answer these questions by choosing from food frequency categories (9 categories in ARIC and FHS, with 1 indicating almost never, whilst 9 indicated ≥6 serves per day; 6 categories in CHS, with 1 indicating never whilst 6 indicated almost every day). These categorical answers were converted to average serves per week for analysis (Additional file 2: Table S2). Serum urate levels (μmolL^−1^) were also measured at the time of data collection (ARIC Visit 1: 1987–1989; uricase oxidation method [31]; CHS Baseline collection: 1989–1990; measured with a Kodak Ektachem 700 analyser with reagents [32]; FHS Generation 3 Exam 1: 2002–2005, auto-analysed with a phosphotungstic acid reagent [33]).

European sample sets for analysis were developed for each of the ARIC, FHS and CHS data sets following consistent exclusion criteria between groups (Additional file 3: Figure S1). People without serum urate measurements or genome-wide genotyping were excluded along with non-European individuals and individuals with kidney disease, established gout, taking urate-lowering drugs, or on diuretic medication. Quality controls for the food frequency data were also used, with individuals who answered less than 10 % of the food frequency survey excluded, along with individuals whose average daily calorie intake was less than 600 kcal/day and greater than 4200 kcal/day. This left 12,720 individuals for analysis (ARIC: 7517, 52 % female, age range 44–65; CHS: 2151, 59 % female, age range 64–97; and FHS: 3052, 54 % female, age range 19–72). Anthropometric, clinical and dietary information for each analysis cohort are contained in Additional file 4: Table S3.

Multivariate linear regression adjusted for common risk factors (sex, age, body mass index and menopausal status), whole-genome principal component analysis vectors 1 and 2 (PCA1 and PCA2) and average daily calorie intake (kcal/day), along with other known dietary triggers, was conducted and the results for the separate ARIC, CHS and FHS sample sets combined by inverse-variance weighted meta-analysis. The ARIC, CHS and FHS cohorts were further divided into male only (ARIC: 3614, CHS: 877, FHS: 1394) and female only (ARIC: 3903, CHS: 1274, FHS: 1658) subsets, on which multivariate linear regression adjusted for age, body mass index, PCA1, PCA2 and calorie intake (and menopausal status in the women only subsets) was performed and the sample sets again combined by meta-analysis. Multivariate linear regression between serum urate levels and each of the known dietary trigger foods (using the same adjustments as stated previously) was also performed for all individuals and the male and female only subsets (Additional file 5: Table S4). All statistical analysis was performed using R v3.0.2 (http://www.R-project.org/). Meta-analysis using an inverse-variance fixed effect model was performed using the R package “meta 3.1-1” (http://cran.r-project.org/web/packages/meta/).

### Results

**Fig. 1:**
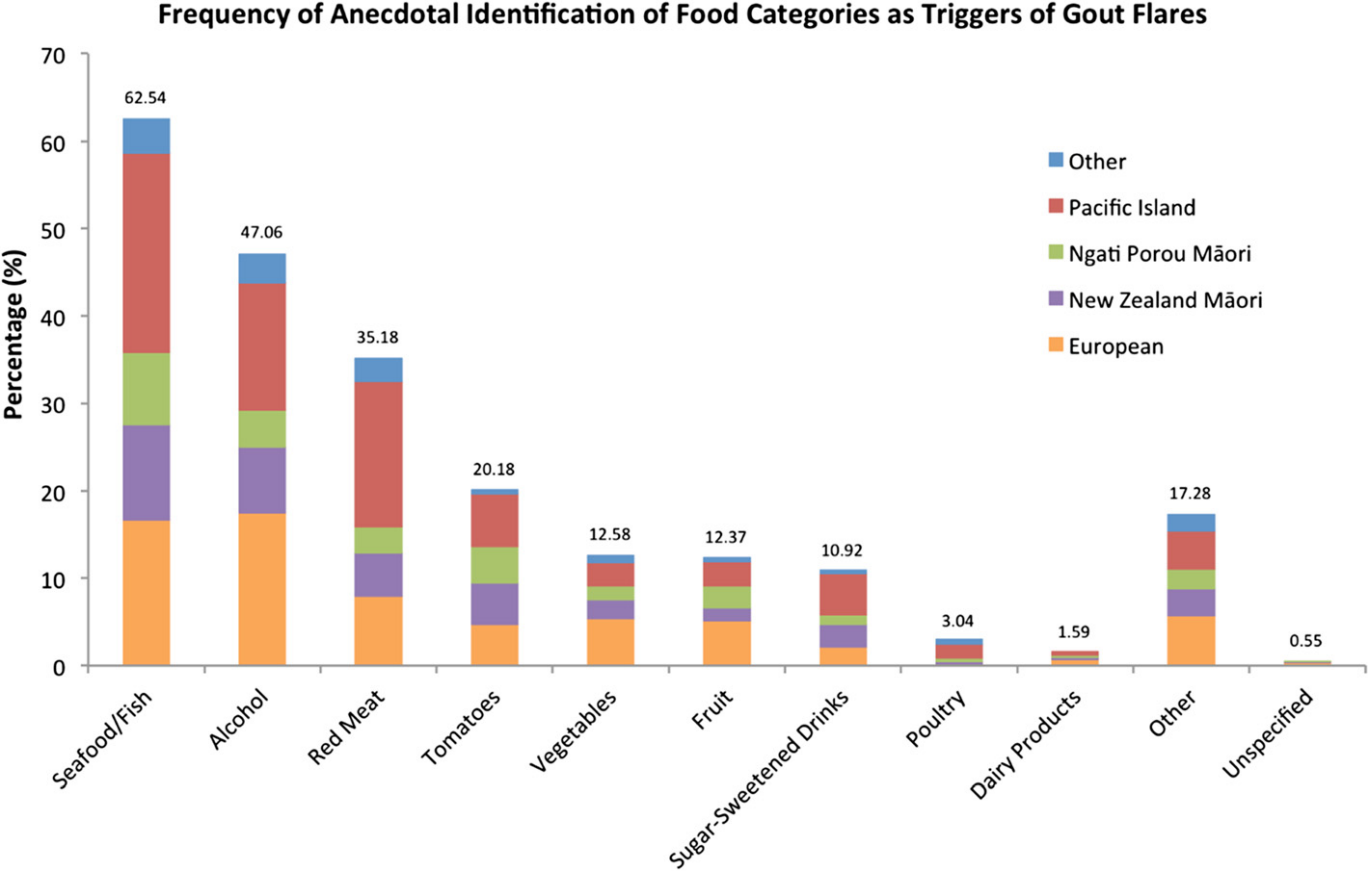
Frequency at which 1447 individuals (478 European, 256 New Zealand Māori, 197 Ngati Porou Māori, 421 Pacific Island, 95 Other ethnicity) who reported ≥1 food trigger of gout flares, specified each food type/category

Of the 2051 New Zealand men and women with gout who were surveyed, 1447 (70.6 %) self-reported ≥1 food or drink trigger of acute gout attacks; 905 (62.5 %) specified seafood or fish, 681 (47.1 %) alcohol, 509 (35.2 %) red meat and 292 (20.2 %) tomatoes (Fig. 1); with 69.4 % specifying >1 trigger. Vegetables, fruit and sugar-sweetened drinks ranked fifth, sixth, and seventh most common reported trigger foods, respectively. Triggers were more commonly reported in New Zealand Māori, Ngati Porou Māori and New Zealand Pacific Island people than European (76.2, 79.1, 88.3 and 57.8 %, respectively). Compared to European, after adjusting for factors influencing the severity of gout, New Zealand Pacific Island people were 3.87-fold (95 % confidence interval (2.72 to 5.61), *P* = 1.9x10^−13^), New Zealand Māori 1.91-fold ((1.38 to 2.68), *P* = 1.3x10^−4^) and Ngati Porou Māori 3.00-fold ((1.98 to 4.66), *P* = 4.7x10^−7^) more likely to self-report a trigger of gout attacks. Similarly Pacific Island people, New Zealand Māori and Ngati Porou Māori were more likely to self-report tomatoes as a trigger of gout flares (1.48-fold ((1.02 to 2.18), *P* = 0.04); 1.98-fold ((1.32 to 2.97), *P* = 8.8x10^−4^); 2.58-fold ((1.69 to 3.93), *P* = 1.0x10^−5^), respectively) than those of European descent.

The three most commonly mentioned dietary triggers of gout have been positively correlated with serum urate levels [12, 15, 25]. Therefore we hypothesised that tomatoes, the fourth most common self-reported trigger of gout flares in New Zealand, also increase serum urate levels. We tested for association between tomato consumption and serum urate in the ARIC, CHS and FHS European Caucasian sample sets. A positive association was observed in the ARIC sample set (β = 0.91 μmolL^−1^ increase in serum urate per additional serve per week of tomatoes, *P* = 0.006) with a positive direction in CHS and FHS (β = 0.22 μmolL^−1^, *P* = 0.79 and β = 0.43 μmolL^−1^, *P* = 0.27 respectively). There was positive association in the combined samples (β = 0.66 μmolL^−1^, *P* = 0.006) (Table 1). The association was also seen in both the men only and women only sub-sets, with a larger effect size in the men only group (men: β = 0.84 μmolL^−1^, *P* = 0.035; women: β = 0.59 μmolL^−1^, *P* = 0.041) (Table 1).

**Table 1:** Association between serum urate levels (μmolL^−1^) and tomato consumption (serves/week)

	All^1^		Men		Women^2^	
	β (95 % CI)	P	β (95 % CI)	P	β (95 % CI)	P
ARIC	0.907 (0.264; 1.550)	0.006	1.399 (0.392; 2.405)	0.006	0.488 ( −0.335; 1.310)	0.245
CHS	0.216 ( −1.341; 1.772)	0.786	1.163 ( −1.578; 3.903)	0.406	−0.279 ( −2.144; 1.586)	0.769
FHS	0.428 ( −0.337; 1.193)	0.273	−0.289 ( −1.665; 1.087)	0.680	0.893 (0.030; 1.757)	0.043
Combined^3^	0.664 (0.194; 1.133)	0.006	0.839 (0.060; 1.618)	0.035	0.592 (0.024; 1.159)	0.041

Adjusted for age, BMI, average calorie intake (kcal/day) and PCA vectors 1 and 2

*P*-value: All = 0.540, Men = 0.148 and Women = 0.505, respectively

^1^Also adjusted for sex and menopause status

^2^Also adjusted for menopause status

^3^Heterozygosity

The association between tomato consumption and serum urate levels was maintained after adjustment for other accepted food triggers, both in a univariate model (red meat: β = 0.69 μmolL^−1^ per additional serving of tomatoes per week, *P* = 0.008; seafood/fish: β = 0.58 μmolL^−1^, *P* = 0.029; sugar-sweetened soft drinks/juices: β = 0.72 μmolL^−1^, *P* = 0.006; dairy products: β = 0.60 μmolL^−1^, *P* = 0.019; coffee: β = 0.67 μmolL^−1^, *P* = 0.011; vitamin C: β = 0.78 μmolL^−1^, *P* = 0.004; alcohol: β = 0.53 μmolL^−1^, *P* = 0.024), and a multivariate model (β = 0.66 μmolL^−1^, *P* = 0.008) (Table 2). Data from the separate sample sets are presented in Additional file 5: Table S4.

**Table 2:** Association between serum urate levels (μmolL^−1^) and tomato consumption (serves/week) adjusted for consumption of known serum urate influencing foods in the meta-analysis combined cohort

	All^1^			Men			Women^2^		
Adjusted by	β (95 % CI)	P	Het P	β (95 % CI)	P	Het P	β (95 % CI)	P	Het P
Red Meat	0.689 (0.177; 1.202)	0.008	0.607	0.855 ( −0.030; 1.740)	0.058	0.260	0.633 (0.030; 1.235)	0.040	0.726
Seafood/Fish	0.575 (0.060; 1.091)	0.029	0.485	0.765 ( −0.125; 1.655)	0.092	0.271	0.467 ( −0.139; 1.073)	0.131	0.637
Sugar −Sweetened Beverages	0.719 (0.207; 1.231)	0.006	0.459	0.909 (0.026; 1.792)	0.044	0.225	0.619 (0.017; 1.222)	0.044	0.593
Dairy Products	0.613 (0.102; 1.124)	0.019	0.600	0.689 ( −0.193; 1.572)	0.126	0.345	0.566 ( −0.035; 1.167)	0.065	0.611
Coffee	0.667 (0.154; 1.180)	0.011	0.544	0.854 ( −0.032; 1.740)	0.059	0.262	0.575 ( −0.027; 1.177)	0.061	0.564
Vitamin C	0.776 (0.253; 1.300)	0.004	0.570	0.889 ( −0.004; 1.782)	0.051	0.284	0.743 (0.124; 1.362)	0.019	0.797
Alcohol	0.534 (0.069; 1.000)	0.024	0.527	0.657 ( −0.114; 1.428)	0.095	0.089	0.468 ( −0.096; 1.032)	0.104	0.487
All Urate Influencing Foods	0.655 (0.173; 1.136)	0.008	0.332	0.665 ( −0.124; 1.45)	0.099	0.105	0.632 (0.045; 1.219)	0.035	0.779

Adjusted for age, BMI, average calorie intake (kcal/day) and PCA vectors 1 and 2

^1^Also adjusted for sex and menopause status

^2^Also adjusted for menopause status

The nutritional exposure data for the specific food items used in this study have previously been validated for the separate ARIC, CHS and FHS cohorts [34–37]. To empirically investigate the validity of the nutritional exposure data we tested four specific foods accepted as urate-raising and conferring of risk of gout (red meat, seafood/fish, sugar-sweetened beverages, alcohol) [9–13] and one urate-lowering and gout protective (dairy products) [11, 12]. Each of these foods was associated with urate, in the expected direction, in either the combined (seafood/fish, dairy products, alcohol) or sex-specific (red-meat, sugar-sweetened beverages) analysis (Additional file 6: Table S5).

## Discussion

Tomatoes are the fourth most common self-reported trigger of gout attacks in a New Zealand sample set. New Zealand Pacific Island, New Zealand Māori and Ngati Porou Māori people were more likely to self-report a trigger of gout flares than European Caucasian (3.87, 1.91 and 3.00-fold, respectively). This was independent of indicators of severity, including number of acute attacks per year. It is possible that self-recognition of attack triggers is a reflection of a greater community familiarity with gout due to a higher prevalence and longer disease history in Pacific Island and Māori populations, with evidence for gout in these populations pre-Westernisation [38, 39]. Tomato has also previously been reported as a food avoided by Australian gout patients, with the authors noting that the current evidence does not support this avoidance [26].

Here we provide evidence that tomato consumption is positively associated with serum urate in European Caucasians suggesting that the avoidance of tomatoes by gout patients (due to identification as triggers of acute gout attacks) is not an unfounded practice. Whilst our data cannot support the claim that tomato consumption is a trigger of gout attacks we provide support for the hypothesis that tomato consumption may trigger gout attacks through increasing serum urate. In order to assess the clinical relevance of these data it is useful to compare the increase in serum urate attributed to a one serve per week increase in tomato consumption (0.7 μmolL^−1^) with other recognised gout attack trigger foods that also increase serum urate (men and women combined). Alcohol increased serum urate by 2.3 μmolL^−1^ per serving per week in the United States third National Health and Nutrition Examination Survey (NHANES) [25]. In the ARIC sample set sugar-sweetened drinks increased urate 0.4 μmolL^−1^ per serve per week [9], and in the third NHANES sample set total meat and seafood increased urate 0.5 μmolL^−1^ and 2.4 μmolL^−1^ per extra serve per week, respectively [12]. Thus tomatoes alter serum urate levels to an extent comparable to other established dietary risk factors for gout.

To assess the causal influence of tomatoes on serum urate levels intervention studies need to be conducted. Small studies investigating the influence of tomatoes or tomato products on other biomarkers have been conducted, with several measuring urate levels before and after intervention took place (Table 3) [40–46]. Lee et al. [43] found an average increase of 46 μmolL^−1^ (*P* < 0.05) in plasma urate levels in a small cohort of young Chinese men (*n* = 10, 26 ± 1 years) 48 h after consumption of 150 g tomato sauce [43]. Conversely a recent study by Vinha et al. [46] found an average drop of 10 μmolL^−1^ in plasma urate levels (*P* < 0.05) in a cohort of young women (*n* = 35, 19.6 ± 1.3 years) who ingested a ~90 g tomato before their midday meal for 30 consecutive days. Four additional intervention studies were unable to find any significant difference in serum or plasma urate levels after intervention [40, 41, 44, 45]. Similarly Jacob et al. [43] found no significant difference in the plasma urate levels of 12 individuals (23 ± 2 years) after consumption of 500 mL tomato juice per day for 2 weeks, however an increase in renal urate clearance (urate:creatinine ratio) was observed in these individuals (*P* < 0.05). Only the study conducted by Vinha et al. [46] used whole tomato fruit as the intervention product, more commonly tomato juice or sauce was used in these studies. This creates the possibility that other additives or the concentrated nature or the processing (eg production and loss of urate-influencing chemicals) of these products influence urate levels, such as sugars (2-3 % content) or added vitamin C (0-4 % content). Generally, the demographics of the groups studied (Table 3) are different (younger) to the group in which we associated tomato consumption with serum urate levels (Additional file 4: Table S3). In the case of Vinha et al. [46] their conclusion that the changes in biochemical and anthropometric parameters may be due to a decrease in postprandial hunger suggests the decrease in plasma urate levels observed could be due to a decrease in consumption of other serum urate-raising foods between meals rather than solely the function of tomato consumption itself. The influence of other serum urate-raising foods was accounted for in our analysis.

**Table 3:** Summary information for seven tomato intervention studies that measured urate levels before and after intervention

Study	Total (n)			Age^1^ (years)	Urate Measurement	Start Urate^2^ (μmolL-1)	End Urate^2^ (μmolL-1)	*P*-value^3^	Intervention
	All	M	F						
Engelhard et al. (37)^4^	31	18	13	48 (30–73)	Serum	336.7 ± 14.3	349.2 ± 15.5	*P* > 0.05	Participants consumed 1 250 mg ‘Lyc-O-Mato’ tomato extract capsule per day for 8 weeks
Jacob et al. (38)	24	4	20	23 (19–27)	Plasma	221.4 ± 49.8	221.0 ± 45.9	*P* > 0.05	Participants consumed 250 mL tomato juice twice daily for 2 weeks^5^
					Urinary (mg/mg Cr)	0.39 ± 0.2	0.52 ± 0.2	*P* < 0.05	
Lee et al. (39)^6^	10	10	-	26	Plasma	348 ± 55	394 ± 40^7^	*P* < 0.01	Participants consumed a single 150 g portion of tomato sauce
Todd et al. (40)^4,8^	34	13	21	52 (27–64)	Plasma	326.1 ± 73.9	329.1 ± 77.5	*P* > 0.05	Participants consumed 500 mL tomato juice per day for 4 weeks
Todd et al. (41)^8^	23	5	18	44 (24–61)	Plasma	280.5 ± 72.0	292.2 ± 67.0	*P* > 0.05	Participants consumed 500 mL tomato juice per day for 4 weeks
Abete et al. (36)	30	18	12	(18–50)	Serum	267.7 ± 53.5	261.7 ± 71.4	*P* > 0.05	Participants consumed 160 g tomato sauce per day for 4 weeks
Vinha et al. (42)	35	-	35	20 (18–25)	Plasma	207.6 ± 55.9	198.1 ± 46.4	*P* < 0.001	Participants consumed an ~90 g tomato each day before lunch for 4 weeks

^1^Average (range)

^2^Average ± standard deviation

^3^All *P*-values were obtained using a Paired Student’s *t*-test or one-way ANOVA to assess data for significant changes before and after intervention (*P* < 0.05 indicates significance)

^4^All participants had hypertension

^5^Participants were divided into two groups – data are shown for only 12 individuals (no demographic data were available)

^6^All participants were of Chinese ethnicity

^7^Measurement taken 48 h after tomato consumption

^8^All uric acid data was not published, data shown here was provided by the study authors directly

The association we have seen between tomatoes and serum urate supports the hypothesis of a possible effect of tomatoes on either hepatic urate production or renal uric acid handling. Any effect of tomatoes on renal uric acid excretion could be mediated by phenolic acid. The inhibitory effects of nine phenolic acid compounds on three renally expressed organic anion transporters (OAT1, OAT3, and OAT4) have been tested [47], all of which transport uric acid [48]. All compounds modified the capability of OAT1 and OAT3 to transport endogenous substrates *in vitro*, whilst two compounds (syringic acid and sinapinic acid) weakly affected OAT4 [47]. Some of the OAT1/OAT3 interacting compounds (*p*-coumaric acid, ferullic acid, *p*-hydroxybenzoic acid and vanillic acid) are present in tomatoes [49]. Of these three transporters, only OAT4 (encoded by *SLC22A11*) has been genetically associated with serum urate and gout [4, 5, 8, 29].

Alternatively tomatoes may be influencing urate levels through increased production. Urate is the end product of purine break-down in humans, and consumption of foods with a high purine-content (red meats, seafood, beer) results in raised serum urate levels and risk of gout [10–12, 25]. Tomatoes, however, have a very low purine-content [50]. Instead tomatoes contain high levels of glutamate, an amino acid which is often found in foods with a high purine-content and is able to stimulate or amplify the synthesis of urate by acting as a nitrogen donor in the purine synthesis pathway [50, 51].

How an increase in serum urate levels could cause an attack of gout is currently not resolved. Intra-articular injection of urate does not induce inflammatory arthritis as compared to monosodium urate (MSU) crystal injection [52] and a well-established trigger of gout attacks is a decrease in urate, for example from initiation of urate-lowering therapy, believed to be caused by crystal shedding in the joint. However, all established dietary triggers of gout flares are characterised by a positive association between consumption and serum urate [12, 15, 25], suggesting that dietary-induced increases in serum urate are important in gout attacks. Given, however, that MSU nucleation and crystal growth is slow compared to dissolution [53] it is possible that the observed dietary-associations with serum urate may require other factors to induce acute gout attacks, for example an increase in free-fatty acids that could be within or induced by the trigger food [54].

To our knowledge this is the first time tomatoes have been associated with serum urate, suggesting that the avoidance of tomatoes by people with gout may have a biological basis. Further research into the relationship between gout (and onset of gout attacks) and tomatoes needs to be conducted, to further investigate this relationship, potentially with a case-crossover study design, as previously used to demonstrate a purine-rich diet and alcohol as triggers of acute gout attacks [22, 24]. Analysis of any effect on urinary uric acid excretion and glutamate metabolism is also required to identify the mechanisms behind this association.

## Conclusion

The positive association between tomato consumption and serum urate levels supports the hypothesis that the self-reporting of tomatoes as a dietary trigger by people with gout has a biological basis.

## Additional files

Additional file 1: Table S1. Characteristics of New Zealand gout study samples. (DOC 65 kb) Additional file 2: Table S2. ARIC, CHS and FHS food frequency questionnaire answer categories and conversion to serves/week. (DOC 37 kb) Additional file 3: Figure S1. Schematic of exclusions for the Atherosclerosis Risk in Communities (ARIC), Cardiovascular Health Study (CHS) and Framingham Heart Study (FHS). (DOC 132 kb) Additional file 4: Table S3. Characteristics of publically-available European study samples. (DOC 57 kb) Additional file 5: Table S4. Association between serum urate levels (μmolL ^−1^) and tomato consumption (serves/week) adjusted for consumption of known serum urate influencing foods in the ARIC, CHS and FHS cohorts. (DOC 71 kb) Additional file 6: Table S5. Association between serum urate levels (μmolL −1) and consumption of five established urate influencing foods (serves/week) in the ARIC, CHS, FHS and combined cohorts. (DOC 66 kb)

## Abbreviations

ARIC: Atherosclerosis Risk in Communities
CHS: Cardiovascular Health Study
FHS: Framingham Heart Study
MSU: Monosodium urate
NHANES: National Health and Nutrition Examination Survey
OAT: Organic anion transporter
PCA: Principal component analysis
SLC: Solute carrier
