# Supplementary material for: Positive association of tomato consumption with serum urate: support for tomato consumption as an anecdotal trigger of gout flares
Source: BMC Musculoskelet Disord. 2015 Aug 19;16:196. doi: 10.1186/s12891-015-0661-8 (PMC4541734; doi:10.1186/s12891-015-0661-8)
Supplement: Additional file 1: Table S1. — Characteristics of New Zealand gout study samples. (DOC 65 kb) [file 12891_2015_661_MOESM1_ESM.doc]

Table S1: Characteristics of New Zealand gout study samples

|  |  | New Zealand Māori |  | Ngati Porou Māori |  | Pacific Island |  | European |  | Other |
| --- | --- | --- | --- | --- | --- | --- | --- | --- | --- | --- |
| Total |  | 343 |  | 260 |  | 486 |  | 834 |  | 128 |
| Gender^1^ |  | 72 (20.99) |  | 45 (17.31) |  | 67 (13.79) |  | 143 (17.15) |  | 13 (10.16) |
| Age (Years) |  | 56.24 ± 12.45 |  | 58.45 ± 12.60 |  | 49.94 ± 13.14 |  | 64.38 ± 12.83 |  | 53.38 ± 14.38 |
| BMI (kg/m^2^) |  | 35.23 ± 8.02 |  | 36.42 ± 8.42 |  | 36.95 ± 8.06 |  | 30.32 ± 6.79 |  | 28.32 ± 4.94 |
| Serum Uric Acid  (μmolL^-1^) |  | 413.36 ± 118.07 |  | 432.34 ± 109.59 |  | 455.51 ± 109.52 |  | 404.83 ± 115.30 |  | 417.42 ± 112.96 |
| Gout Characteristics | | | | | | | | | | |
| Duration of Gout (Years) |  | 15.33 ± 12.88 |  | 18.36 ± 14.91 |  | 12.15 ± 11.11 |  | 15.18 ± 13.04 |  | 10.36 ± 10.89 |
| Age at First Attack (Years) |  | 40.73 ± 15.74 |  | 40.62 ± 15.26 |  | 37.71 ± 12.98 |  | 49.24 ± 16.87 |  | 43.19 ± 13.37 |
| Number of Attacks Per Year |  | 10.55 ± 41.28 |  | 2.96 ± 4.96 |  | 9.40 ± 27.30 |  | 6.97 ± 34.39 |  | 8.24 ± 33.12 |
| Taking Urate Lowering Drugs^2^ |  | 277 (80.76) |  | 191 (73.46) |  | 379 (77.98) |  | 596 (71.46) |  | 95 (74.22) |
| Gout Attack Triggers | | | | | | | | | | |
| Number of Trigger Categories Mentioned |  | 1.70 ± 1.41 |  | 1.76 ± 1.27 |  | 2.51 ± 1.49 |  | 1.21 ± 1.26 |  | 1.73 ± 1.42 |
| Seafood/Fish^3^ |  | 157 (59.70) |  | 121 (59.02) |  | 329 (77.41) |  | 241 (49.39) |  | 57 (60.00) |
| Alcohol^3^ |  | 109 (41.13) |  | 60 (29.56) |  | 212 (49.77) |  | 253 (51.11) |  | 47 (48.96) |
| Red Meat^3^ |  | 72 (28.13) |  | 44 (22.34) |  | 240 (57.01) |  | 114 (23.80) |  | 39 (41.05) |
| Tomatoes^3^ |  | 68 (26.56) |  | 61 (30.96) |  | 87 (20.67) |  | 68 (14.23) |  | 8 (8.42) |
| Vegetables^3^ |  | 31 (12.11) |  | 23 (11.68) |  | 38 (9.03) |  | 78 (16.32) |  | 12 (12.63) |
| Fruit^3^ |  | 21 (8.20) |  | 37 (18.78) |  | 40 (9.50) |  | 74 (15.48) |  | 7 (7.37) |
| Sugar-Sweetened Beverages^3^ |  | 37 (14.45) |  | 16 (8.12) |  | 68 (16.15) |  | 31 (6.49) |  | 6 (6.32) |
| Poultry^3^ |  | 4 (1.56) |  | 5 (2.54) |  | 24 (5.70) |  | 3 (0.63) |  | 8 (8.42) |
| Dairy Products^3^ |  | 4 (1.56) |  | 3 (1.52) |  | 6 (1.43) |  | 10 (2.09) |  | 0 (0.00) |
| Other^3^ |  | 44 (17.19) |  | 32 (16.24) |  | 63 (14.96) |  | 83 (17.36) |  | 28 (29.47) |

^1^Displayed as total number of females and percentage females; n (%)

^2^Displayed as total number of individuals on urate lowering drugs (Allopurinol, Probenecid, Benzbromarone and Febuxostat) and percentage; n (%)

^3^Displayed as total number and percentage out of the total number of individuals who identified ≥1 gout attack trigger food; n (%)
