# Supplementary material for: Positive association of tomato consumption with serum urate: support for tomato consumption as an anecdotal trigger of gout flares
Source: BMC Musculoskelet Disord. 2015 Aug 19;16:196. doi: 10.1186/s12891-015-0661-8 (PMC4541734; doi:10.1186/s12891-015-0661-8)
Supplement: Additional file 2: Table S2. — ARIC, CHS and FHS food frequency questionnaire answer categories and conversion to serves/week. (DOC 37 kb) [file 12891_2015_661_MOESM2_ESM.doc]

Table S2: ARIC, CHS and FHS food frequency questionnaire answer categories and conversion to serves/week

| No. |  | ARIC | |  | CHS^1^ | |  | FHS | |
| --- | --- | --- | --- | --- | --- | --- | --- | --- | --- |
|  |  | Category | Serves/week conversion |  | Category | Serves/week conversion |  | Category | Serves/week conversion |
| 1 |  | Almost never | 0 |  | Never | 0 |  | Never, or <1 per month | 0 |
| 2 |  | 1-3 serves per month | 0.47 |  | 5-10 times per year | 0.14 |  | 1-3 serves per month | 0.47 |
| 3 |  | 1 serve per week | 1 |  | 1-3 times per month | 0.47 |  | 1 serve per week | 1 |
| 4 |  | 2-4 serves per week | 3 |  | 1-4 times per week | 2.5 |  | 2-4 serves per week | 3 |
| 5 |  | 5-6 serves per week | 5.5 |  | Almost every day | 6 |  | 5-6 serves per week | 5.5 |
| 6 |  | 1 serve per day | 7 |  |  |  |  | 1 serve per day | 7 |
| 7 |  | 2-3 serves per day | 17.5 |  |  |  |  | 2-3 serves per day | 17.5 |
| 8 |  | 4-6 serves per day | 35 |  |  |  |  | 4-5 serves per day | 31.5 |
| 9 |  | >6 serves per day | 42 |  |  |  |  | 6+ serves per day | 42 |

^1^CHS categories were concerned with frequency of consumption, serving size was not specified, consumption multiple times per day was not considered
