# Supplementary material for: Positive association of tomato consumption with serum urate: support for tomato consumption as an anecdotal trigger of gout flares
Source: BMC Musculoskelet Disord. 2015 Aug 19;16:196. doi: 10.1186/s12891-015-0661-8 (PMC4541734; doi:10.1186/s12891-015-0661-8)

Figure S1: Schematic of exclusions for the Atherosclerosis Risk in Communities (ARIC), Cardiovascular Health Study (CHS) and Framingham Heart Study (FHS).

^1^CHS participants were not asked about gout status.

^2^ARIC: where multiple individuals from a family were collected all but one of these individuals were removed. CHS: several individuals had been collected for other studies previously (ARIC, SHEP or NHANES3) these individuals were removed to avoid overlap. FHS had no study-specific removal criteria.


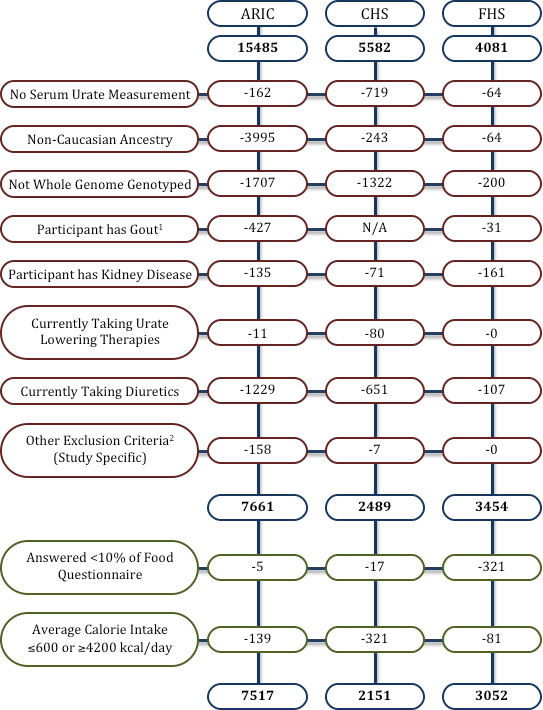

Supplement: Additional file 3: Figure S1. — Schematic of exclusions for the Atherosclerosis Risk in Communities (ARIC), Cardiovascular Health Study (CHS) and Framingham Heart Study (FHS). (DOC 132 kb) [file 12891_2015_661_MOESM3_ESM.doc]
