# Supplementary material for: Positive association of tomato consumption with serum urate: support for tomato consumption as an anecdotal trigger of gout flares
Source: BMC Musculoskelet Disord. 2015 Aug 19;16:196. doi: 10.1186/s12891-015-0661-8 (PMC4541734; doi:10.1186/s12891-015-0661-8)
Supplement: Additional file 4: Table S3. — Characteristics of publically-available European study samples. (DOC 57 kb) [file 12891_2015_661_MOESM4_ESM.doc]

Table S3: Characteristics of publically-available European study samples

|  |  | ARIC |  | CHS |  | FHS |
| --- | --- | --- | --- | --- | --- | --- |
| Total |  | 7517 |  | 2151 |  | 3052 |
| Gender^1^ |  | 3903 (51.92) |  | 1274 (59.23) |  | 1658 (54.33) |
| PreMenopausal^1^ |  | 640 (16.40) |  | - |  | 1395 (84.14) |
| PostMenopausal^1^ |  | 1999 (51.22) |  | 1136 (89.17) |  | 210 (12.67) |
| Age (Years) |  | 53.99 ± 5.64 |  | 72.21 ± 5.37 |  | 39.85 ± 8.76 |
| BMI (kg/m^2^) |  | 26.46 ± 4.50 |  | 25.84 ± 4.14 |  | 26.51 ± 5.18 |
| Serum Uric Acid (μMolL^-1^) |  | 338.60 ± 80.74 |  | 316.19 ± 79.30 |  | 307.55 ± 85.48 |
| PCA1 |  | -0.00499 ± 0.00043 |  | 0.00658 ± 0.00117 |  | -0.00043 ± 0.00974 |
| PCA2 |  | 0.00008 ± 0.00858 |  | 0.00090 ± 0.01774 |  | -0.00040 ± 0.01327 |
| Average Calorie Intake (kcal/day) |  | 1,640.78 ± 603.91 |  | 1,814.50 ± 641.46 |  | 2,070.87 ± 689.07 |
| Vitamin C (mg/day) |  | 116.86 ± 76.84 |  | 197.00 ± 93.54 |  | 227.78 ± 286.04 |
| Estimated Dietary Intake (Servings/Week) | | | | | | |
| Tomatoes |  | 1.98 ± 2.35 |  | 1.83 ± 1.95 |  | 2.51 ± 2.85 |
| Red Meat |  | 7.62 ± 5.67 |  | 6.17 ± 5.24 |  | 5.60 ± 4.23 |
| Seafood/Fish |  | 2.02 ± 2.06 |  | 3.22 ± 2.72 |  | 2.25 ± 2.16 |
| Sugar-Sweetened Beverages |  | 6.93 ± 7.25 |  | 5.33 ± 3.74 |  | 8.86 ± 9.37 |
| Dairy Products |  | 23.90 ± 14.57 |  | 16.70 ± 7.83 |  | 24.58 ± 15.41 |
| Coffee |  | 14.57 ± 14.33 |  | 2.98 ± 2.82 |  | 9.15 ± 9.26 |
| Estimated Alcohol Intake (Servings/Week) | | | | | | |
| Alcohol |  | 3.42 ± 6.83 |  | 2.85 ± 6.52 |  | 5.60 ± 7.55 |
| Beer |  | 1.59 ± 5.08 |  | 0.77 ± 3.64 |  | 2.75 ± 5.57 |
| Wine |  | 0.62 ± 1.85 |  | 0.69 ± 2.33 |  | 2.04 ± 3.76 |
| Liquor |  | 1.21 ± 3.43 |  | 1.40 ± 4.24 |  | 0.83 ± 2.54 |

^1^Displayed as total number of females and percentage females; n (%)

^2^Displayed as total number and percentage out of total number of females; n (%
