# Supplementary material for: Positive association of tomato consumption with serum urate: support for tomato consumption as an anecdotal trigger of gout flares
Source: BMC Musculoskelet Disord. 2015 Aug 19;16:196. doi: 10.1186/s12891-015-0661-8 (PMC4541734; doi:10.1186/s12891-015-0661-8)
Supplement: Additional file 5: Table S4. — Association between serum urate levels (μmolL −1) and tomato consumption (serves/week) adjusted for consumption of known serum urate influencing foods in the ARIC, CHS and FHS cohorts. (DOC 71 kb) [file 12891_2015_661_MOESM5_ESM.doc]

Table S4: Association between serum urate levels (μmolL^-1^) and tomato consumption (serves/week) adjusted for consumption of known serum urate influencing foods in the ARIC, CHS and FHS cohorts.

|  |  |  |  | All^1^ | |  | Men | |  | Women^2^ | |
| --- | --- | --- | --- | --- | --- | --- | --- | --- | --- | --- | --- |
| Adjusted by |  | Study |  | β [95% CI] | P |  | β [95% CI] | P |  | β [95% CI] | P |
| Red Meat |  | ARIC |  | 0.911  [0.268; 1.554] | 0.006 |  | 1.400  [0.394; 2.406] | 0.006 |  | 0.521  [-0.302; 1.344] | 0.215 |
|  |  | CHS |  | 0.318  [-1.248; 1.883] | 0.691 |  | 1.121  [-1.630; 3.871] | 0.425 |  | -0.001  [-1.877; 1.876] | 0.999 |
|  |  | FHS |  | 0.444  [-0.323; 1.211] | 0.256 |  | -0.268  [-1.648; 1.111] | 0.703 |  | 0.909  [0.000; 0.043] | 0.040 |
| Seafood/Fish |  | ARIC |  | 0.869  [0.221; 1.517] | 0.009 |  | 1.346  [0.333; 2.359] | 0.009 |  | 0.457  [-0.372; 1.286] | 0.280 |
|  |  | CHS |  | 0.143  [-1.417; 1.703] | 0.857 |  | 1.143  [-1.599; 3.885] | 0.414 |  | -0.390  [-2.263; 1.484] | 0.684 |
|  |  | FHS |  | 0.286  [-0.486; 1.058] | 0.468 |  | -0.364  [-1.752; 1.023] | 0.607 |  | 0.687  [-0.185; 1.559] | 0.123 |
| Sugar-Sweetened Beverages |  | ARIC |  | 0.992  [0.350; 1.634] | 0.002 |  | 1.465  [0.461; 2.469] | 0.004 |  | 0.570  [-0.253; 1.393] | 0.174 |
|  |  | CHS |  | 0.221  [-1.336; 1.778] | 0.781 |  | 1.161  [-1.581; 3.903] | 0.407 |  | -0.271  [-2.137; 1.595] | 0.776 |
|  |  | FHS |  | 0.443  [-0.323; 1.209] | 0.257 |  | -0.265  [-1.641; 1.112] | 0.707 |  | 0.893  [0.000; 0.026] | 0.044 |
| Dairy Products |  | ARIC |  | 0.831  [0.189; 1.473] | 0.011 |  | 1.201  [0.197; 2.206] | 0.019 |  | 0.482  [-0.340; 1.303] | 0.250 |
|  |  | CHS |  | 0.185  [-1.372; 1.742] | 0.816 |  | 1.075  [-1.663; 3.814] | 0.442 |  | -0.279  [-2.146; 1.587] | 0.769 |
|  |  | FHS |  | 0.384  [-0.380; 1.147] | 0.325 |  | -0.333  [-1.706; 1.041] | 0.635 |  | 0.847  [-0.016; 1.710] | 0.055 |
| Coffee |  | ARIC |  | 0.906  [0.263; 1.549] | 0.006 |  | 1.392  [0.386; 2.399] | 0.007 |  | 0.491  [-0.332; 1.313] | 0.243 |
|  |  | CHS |  | 0.168  [-1.391; 1.727] | 0.833 |  | 1.102  [-1.645; 3.848] | 0.432 |  | -0.325  [-2.192; 1.543] | 0.733 |
|  |  | FHS |  | 0.429  [-0.339; 1.197] | 0.273 |  | -0.276  [-1.660; 1.109] | 0.696 |  | 0.876  [0.010; 1.742] | 0.048 |
| Vitamin C |  | ARIC |  | 0.982  [0.315; 1.649] | 0.004 |  | 1.293  [0.269; 2.317] | 0.013 |  | 0.730  [-0.136; 1.596] | 0.099 |
|  |  | CHS |  | 0.583  [-1.003; 2.169] | 0.472 |  | 1.443  [-1.342; 4.228] | 0.310 |  | 0.125  [-1.778; 2.028] | 0.897 |
|  |  | FHS |  | 0.464  [-0.305; 1.232] | 0.237 |  | -0.213  [-1.594; 1.169] | 0.763 |  | 0.893  [0.024; 1.761] | 0.044 |
| Alcohol |  | ARIC |  | 0.789  [0.148; 1.429] | 0.016 |  | 1.255  [0.254; 2.256] | 0.014 |  | 0.359  [-0.461; 1.180] | 0.391 |
|  |  | CHS |  | 0.220  [-1.331; 1.771] | 0.781 |  | 1.306  [-1.432; 4.044] | 0.350 |  | -0.421  [-2.278; 1.437] | 0.657 |
|  |  | FHS |  | 0.258  [-0.494; 1.010] | 0.502 |  | -0.581  [-1.927; 0.765] | 0.397 |  | 0.774  [-0.081; 1.629] | 0.076 |
| All Urate Influencing Foods |  | ARIC |  | 1.000  [0.328; 1.671] | 0.004 |  | 1.239  [0.209; 2.270] | 0.019 |  | 0.741  [-0.134; 1.615] | 0.097 |
|  |  | CHS |  | 0.545  [-1.049; 2.139] | 0.503 |  | 1.490  [-1.318; 4.298] | 0.299 |  | -0.014  [-1.929; 1.901] | 0.989 |
|  |  | FHS |  | 0.232  [-0.533; 0.997] | 0.552 |  | -0.539  [-1.906; 0.828] | 0.440 |  | 0.658  [-0.214; 1.529] | 0.139 |

Also adjusted for age, BMI, average calorie intake (kcal/day) and PCA vectors 1 and 2

^1^Also adjusted for sex and menopause status

^2^Also adjusted for menopause status
