# Supplementary material for: Positive association of tomato consumption with serum urate: support for tomato consumption as an anecdotal trigger of gout flares
Source: BMC Musculoskelet Disord. 2015 Aug 19;16:196. doi: 10.1186/s12891-015-0661-8 (PMC4541734; doi:10.1186/s12891-015-0661-8)
Supplement: Additional file 6: Table S5. — Association between serum urate levels (μmolL −1) and consumption of five established urate influencing foods (serves/week) in the ARIC, CHS, FHS and combined cohorts. (DOC 66 kb) [file 12891_2015_661_MOESM6_ESM.doc]

Table S5: Association between serum urate levels (μmolL-1) and consumption of five established urate influencing foods (serves/week) in the ARIC, CHS, FHS and combined cohorts.

|  |  | Study |  | All^1^ | |  | Men | |  | Women^2^ | |
| --- | --- | --- | --- | --- | --- | --- | --- | --- | --- | --- | --- |
|  |  |  |  | β [95% CI] | P |  | β [95% CI] | P |  | β [95% CI] | P |
| Red Meat |  | ARIC |  | 0.122 [-0.202; 0.447] | 0.460 |  | -0.056 [-0.496; 0.384] | 0.803 |  | 0.487 [-0.003; 0.977] | 0.051 |
|  |  | CHS |  | 0.486 [-0.289; 1.261] | 0.219 |  | -0.248 [-1.429; 0.933] | 0.681 |  | 1.273 [0.235; 2.311] | 0.016 |
|  |  | FHS |  | 0.199 [-0.398; 0.795] | 0.514 |  | 0.219 [-0.691; 1.129] | 0.637 |  | 0.223 [-0.564; 1.009] | 0.579 |
|  |  | Combined^3^ |  | 0.181 [-0.087; 0.449] | 0.186 |  | -0.029 [-0.404; 0.346] | 0.881 |  | 0.531 [0.145; 0.918] | 0.007 |
| Seafood/Fish |  | ARIC |  | 0.514 [-0.215; 1.243] | 0.167 |  | 0.716 [-0.469; 1.901] | 0.236 |  | 0.390 [-0.517; 1.296] | 0.400 |
|  |  | CHS |  | 0.825 [-0.411; 2.061] | 0.191 |  | 0.682 [-1.535; 2.899] | 0.547 |  | 0.850 [-0.618; 2.319] | 0.257 |
|  |  | FHS |  | 1.337 [0.324; 2.350] | 0.010 |  | 0.612 [-0.989; 2.212] | 0.454 |  | 2.071 [0.801; 3.341] | 0.001 |
|  |  | Combined^3^ |  | 0.801 [0.267; 1.335] | 0.003 |  | 0.680 [-0.196; 1.555] | 0.128 |  | 0.936 [0.276; 1.596] | 0.005 |
| Sugar-Sweetened Beverages |  | ARIC |  | 0.677 [0.452; 0.901] | 3.47E-09 |  | 0.747 [0.421; 1.074] | 7.40E-06 |  | 0.527 [0.220; 0.835] | 0.001 |
|  |  | CHS |  | -0.173 [-1.000; 0.655] | 0.683 |  | 0.023 [-1.327; 1.374] | 0.973 |  | -0.244 [-1.289; 0.801] | 0.647 |
|  |  | FHS |  | 0.088 [-0.172; 0.349] | 0.508 |  | 0.208 [-0.190; 0.606] | 0.306 |  | -0.020 [-0.363; 0.323] | 0.910 |
|  |  | Combined^3^ |  | 0.273 [-0.235; 0.781] | 0.292 |  | 0.512 [0.264; 0.761] | 5.31E-05 |  | 0.186 [-0.281; 0.653] | 0.434 |
| Dairy Products |  | ARIC |  | -0.401 [-0.520; -0.282] | 4.71E-11 |  | -0.496 [-0.666; -0.326] | 1.11E-08 |  | -0.292 [-0.459; -0.125] | 0.001 |
|  |  | CHS |  | -0.273 [-0.727; 0.181] | 0.238 |  | -0.668 [-1.405; 0.070] | 0.076 |  | 0.013 [-0.560; 0.585] | 0.966 |
|  |  | FHS |  | -0.241 [-0.396; -0.086] | 0.002 |  | -0.244 [-0.494; 0.007] | 0.057 |  | -0.253 [-0.445; -0.062] | 0.010 |
|  |  | Combined^3^ |  | -0.339 [-0.431; -0.246] | 7.05E-13 |  | -0.426 [-0.564; -0.288] | 1.52E-09 |  | -0.262 [-0.385; -0.139] | 3.01E-05 |
| Alcohol |  | ARIC |  | 1.026 [0.805; 1.247] | 1.42E-19 |  | 0.922 [0.667; 1.178] | 1.76E-12 |  | 1.484 [0.988; 1.980] | 5.11E-09 |
|  |  | CHS |  | 0.998 [0.536; 1.460] | 2.38E-05 |  | 0.800 [0.204; 1.396] | 0.009 |  | 1.396 [0.615; 2.177] | 4.79E-04 |
|  |  | FHS |  | 1.652 [1.359; 1.945] | 7.51E-28 |  | 1.692 [1.306; 2.078] | 2.08E-17 |  | 1.669 [1.160; 2.178] | 1.71E-10 |
|  |  | Combined^3^ |  | 1.236 [0.796; 1.676] | 3.67E-08 |  | 1.151 [0.600; 1.703] | 4.25E-05 |  | 1.543 [1.219; 1.866] | 9.05E-21 |

Also adjusted for age, BMI, average calorie intake (kcal/day) and PCA vectors 1 and 2

^1^Also adjusted for sex and menopause status

^2^Also adjusted for menopause status

^3^Heterozygosity *P*-value: All*=*0.697, Men=0.806, Women=0.277 for red meat; All=0.431, Men=0.995, Women=0.107 for seafood/fish; All=0.001, Men=0.094, Women=0.042 for sugar-sweetened beverages; All=0.265, Men=0.213, Women=0.602 for dairy products; All=0.003, Men=0.003, Women=0.807 for alcohol.
